# Semi-synthesis and biological activities of heterocyclic compounds containing camphor

**DOI:** 10.1039/d5ra00484e

**Published:** 2025-04-25

**Authors:** Zeinab S. Hamza, Wael N. Hozzein, Khaled El-Adl, Mohammad K. Okla, Gehad AbdElgayed, Wael A. A. Fadaly, Hussein S. Mohamed

**Affiliations:** a Chemistry of Medicinal and Aromatic Plants Department, Research Institute of Medicinal and Aromatic Plants (RIMAB), Beni-Suef University Beni-Suef 62511 Egypt h_gendy_2010@yahoo.com Husseinshaban@rimp.bsu.edu.eg +201000800296; b Botany and Microbiology Department, Faculty of Science, Beni-Suef University Beni-Suef Egypt; c Chemistry Department, Faculty of Pharmacy, Heliopolis University for Sustainable Development Cairo Egypt; d Pharmaceutical Medicinal Chemistry and Drug Design Department, Faculty of Pharmacy (Boys), Al-Azhar University Nasr City 11884 Cairo Egypt; e Botany and Microbiology Department, College of Science, King Saud University P.O. Box 2455 Riyadh 11451 Saudi Arabia; f Integrated Molecular Plant Physiology Research, Department of Biology, University of Antwerp Antwerp 2020 Belgium; g Pharmaceutical Organic Chemistry Department, Faculty of Pharmacy, Beni-Suef University Beni-Suef Egypt

## Abstract

Breast cancer and lung cancer are two of the most prevalent and deadly malignancies worldwide. Both cancers present significant challenges in terms of effective treatment and management, highlighting the urgent need for novel therapeutic strategies that can improve patient outcomes. This study focuses on the synthesis of novel heterocyclic compounds derived from the naturally formed camphor, aimed at evaluating their cytotoxicity. The research addresses the need for effective cancer treatments by presenting compounds that demonstrate significant inhibitory effects against MCF-7 breast carcinoma cells. Among these, compound 20 exhibited remarkable potency, with an IC_50_ value of 0.78 μM, surpassing the efficacy of standard chemotherapeutics, dasatinib (IC_50_ = 7.99 μM) and doxorubicin (IC_50_ = 3.10 μM). In the context of A549 lung cancer cells, compound 20 also showed strong inhibitory activity (IC_50_ = 1.69 μM), again outperforming dasatinib (IC_50_ = 11.8 μM) and doxorubicin (IC_50_ = 2.43 μM). To further elucidate the biological activities of these compounds, molecular docking studies were performed, revealing that compound 20 exhibited the highest binding energy among the tested compounds, supporting the experimental findings. These results indicate that the synthesized camphor-derived heterocycles, particularly compound 20, have significant potential as potent anticancer agents against breast and lung cancer cell lines.

## Abstract 2

Semi-synthesis and molecular docking of camphor derivatives with anti-cancer activity.

## Graphical abstract

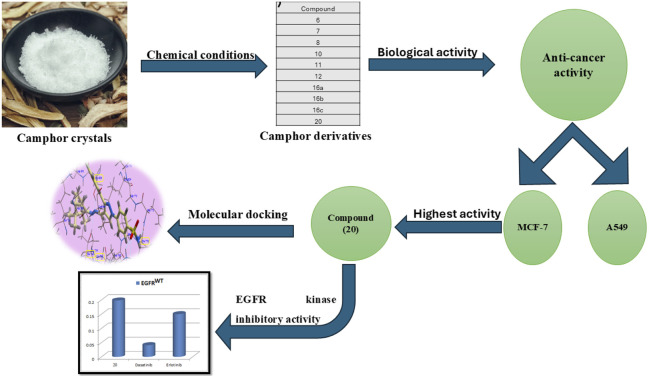

## 1. Introduction

Breast cancer and lung cancer are two prevalent types of cancer that significantly impact the health and well-being of individuals worldwide. Breast cancer is a type of cancer that arises inside the tissue of the breast, typically originating in the milk ducts or lobules of the breast.^1,2^ It is the most prevalent cancer among women worldwide, although it can also affect men.^3,4^ Breast cancer treatment options rely on multiple factors, involving the cancer stage, tumor characteristics, and one's health in general.^5–7^ Standard treatment options encompass surgical procedures (such as lumpectomy or mastectomy), chemotherapy, radiation, targeted therapy, and hormonal therapy.^8–11^ With advancements in early detection and treatment strategies, the outlook for carcinoma of the breast has markedly improved in recent years.^12,13^

A lung tumor is a malignancy that arises in the lungs, typically in the cells that line the airways.^14^ It affects both sexes and is the main reason for cancer-related fatalities globally.^15,16^ The primary cause of lung cancer is tobacco smoke, including second-hand smoke inhalation and cigarettes, cigars, and tobacco pipe smoking.^17–20^ Surgery, therapy with radiation, treatment with chemotherapy, targeted therapy, and immunotherapy constitute typical forms of treatment.^21,22^ Targeted therapies for lung cancer often focus on specific genetic mutations, such as EGFR, ALK, ROS1, and others, which can drive the growth of cancer cells. It is noteworthy that the prognosis for both breast cancer and lung cancer is improved by early detection and prompt medical care.^23^

EGFR (Epidermal Growth Factor Receptor) enzyme inhibition refers to the suppression or blocking of the activity of the EGFR enzyme.^24^ The receptor tyrosine kinase EGFR is important for cell proliferation,^25^ proliferation, and survival.^26^ Abnormal activation or overexpression of EGFR The development and progression of certain cancer types, including as colorectal, lung, and cancers of the neck and head, are frequently linked to it.^27^ Inhibition of EGFR activity can be achieved through different approaches, primarily using targeted therapies known as EGFR inhibitors.^28^ EGFR inhibitors have shown clinical efficacy in certain cancers that exhibit EGFR abnormalities. They are commonly used in EGFR mutation therapy for non-small cell lung cancer (NSCLC) and metastatic colorectal cancer with EGFR overexpression.^29^ These targeted therapies can be used as monotherapy or in conjunction with additional treatment approaches, such radiation therapy or chemotherapy, based on the particular malignancy and its features.

Camphor is a waxy, white, or transparent solid produced from turpentine oil or from the bark that comes from the plant *Cinnamomum camphora*.^30^ It has a distinct, aromatic odor and a cooling sensation when applied to the skin.^31^*C. camphora* has a long history of use in various cultures and is utilized for its medicinal, aromatic, and pest-repellent properties. In traditional medicine, *C. camphora* has been employed for its potential therapeutic effects.^32^*Cinnamomum camphora* is a species endemic to China and is highly valued as a natural spice plant in the country.^33,34^ The leaves of it contain a significant amount of essential oil (3.8–4.5%), consisting of more than 40 components, including 1,8-cineole, γ-terpinene, terpinene-4-ol, α-terpineol, and safrole.^35^ Research has shown that the essential oil derived from the leaves of it exhibits various biological activities, including antimicrobial^36–38^ effects, antitumor actions,^39–41^ and analgesic properties.^42^ In addition to its medicinal uses, *C. camphora* has been utilized as an aromatic ingredient in perfumes, soaps, and other personal care products.^43^ Overall, *C. camphora* is a natural plant with a rich history of traditional use.^30^

Thiazoles are an important group of compounds due to their diverse chemical and biological properties.^44^ Their chemical structure and reactivity make them versatile building blocks for the synthesis of complex organic molecules.^45,46^ Thiazole derivatives often exhibit interesting biological activities, such as antimicrobial, antiviral, anticancer, and anti-inflammatory properties.^47^ Studies have shown that certain thiazole derivatives exhibit promising anticancer activity against breast and lung cancer cells *in vitro* (in cell cultures) and *in vivo* (in animal models).^48^

Prymidinone and pyrimidinthione are synthetic compounds that belong to the class of pyrimidine derivatives.^49,50^ Several derivatives are being explored for their ability to inhibit specific kinases involved in cancer progression, such as The receptors for vascular endothelial growth factor (VEGFR) and epidermal growth factor (EGFR).^51,52^ These inhibitors may enhance targeted cancer therapies.

Pyrazole derivatives have emerged as a promising class of compounds in cancer research due to their diverse biological activities, including anti-cancer properties.^53,54^ Many pyrazole derivatives can trigger cancer cells that undergo programmed cell death, promoting apoptosis and thereby reducing tumor size and preventing metastasis.^52,55^ Some pyrazole derivatives function as kinase inhibitors, targeting specific enzymes involved in tumor growth and survival, such as EGFR, VEGFR, and CDK. This makes them valuable in targeted cancer therapies.^56^

Dasatinib is a potent and selective tyrosine kinase inhibitor (TKI) primarily utilized to treat some cancers, including acute lymphoblastic leukemia (ALL) and chronic myeloid leukemia (CML).^57^ The U.S. Food and Drug Administration (FDA) gave its approval in 2006,^58^ Dasatinib was designed to suppress the BCR-ABL fusion protein's activity, which is a characteristic of leukemias with the Philadelphia chromosome.^59^

Doxorubicin, also known as Adriamycin, is a widely used anthracycline antibiotic and chemotherapeutic agent that is essential to the treatment of many types of cancer.^60^ First isolated from the bacterium *Streptomyces peucetius* in the 1960s,^61^ Doxorubicin has since become a cornerstone in oncology due to its broad-spectrum efficacy against solid tumors and hematological malignancies.^62^

**Fig. 1:**
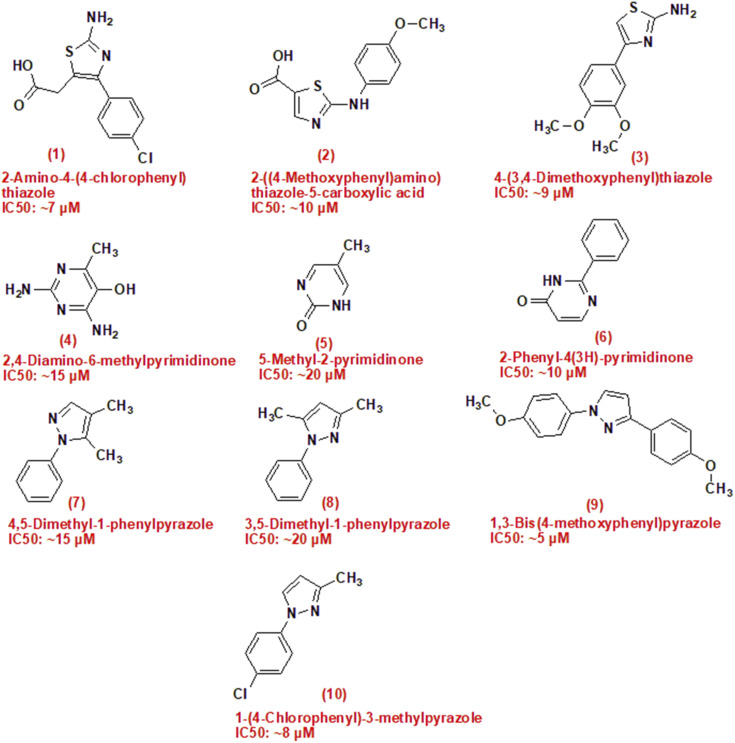
Structure of compounds has anticancer activities, (1–3) are thiazole derivatives, (4–6) are pyrimidinone derivatives and (7–10) are pyrazole derivatives.

In summary, the pressing challenges posed by breast and lung cancer necessitate the continued exploration of novel compounds, such as camphor-derived heterocycles, to enhance treatment efficacy and improve patient outcomes. This study aims to contribute to this effort by investigating the potential cytotoxicity of newly synthesized compounds (Fig. 1).

## 2. Rationale and structure design

**Fig. 2:**
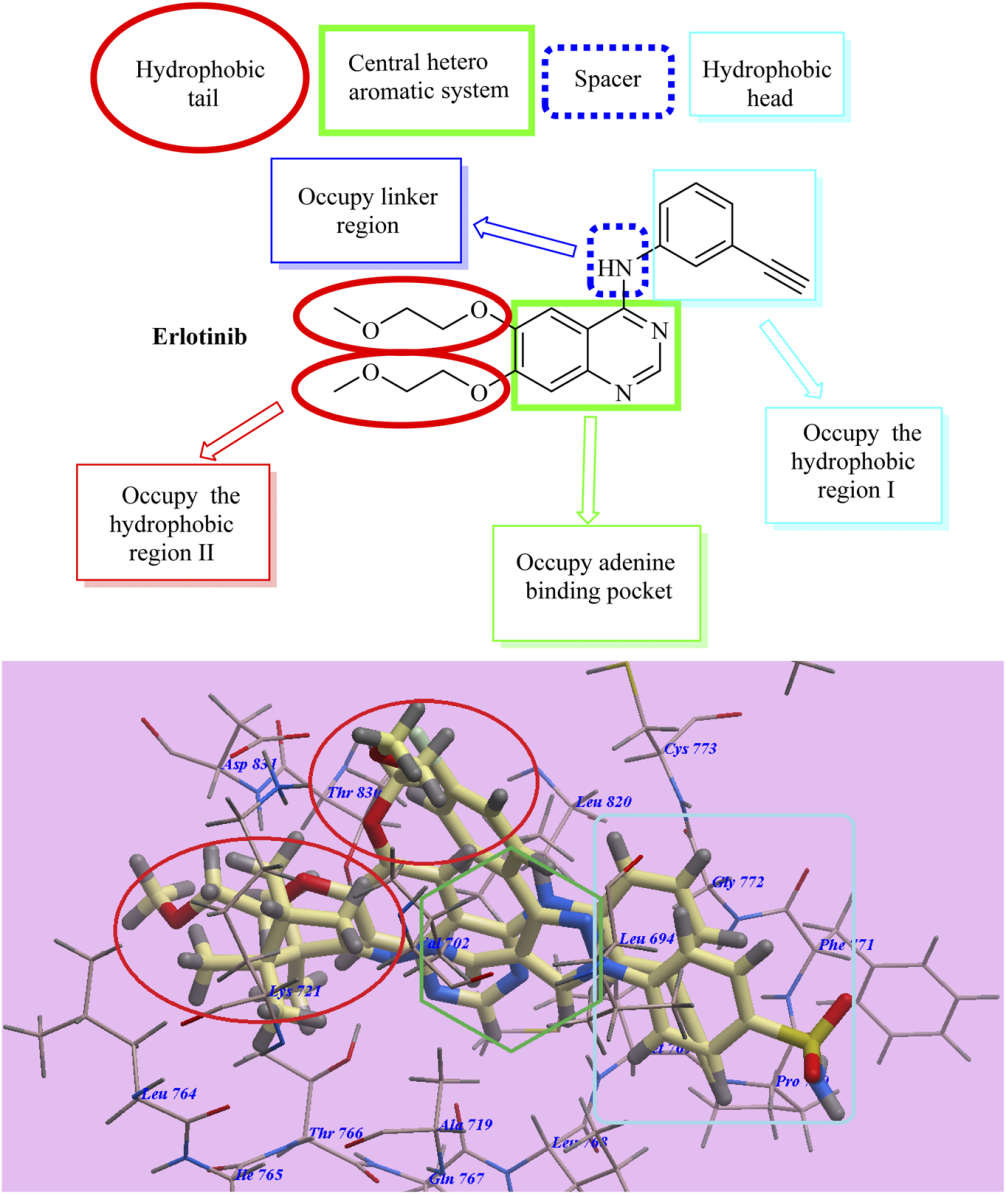
Essential necessities for erlotinib as reference TK inhibitor.

**Fig. 3:**
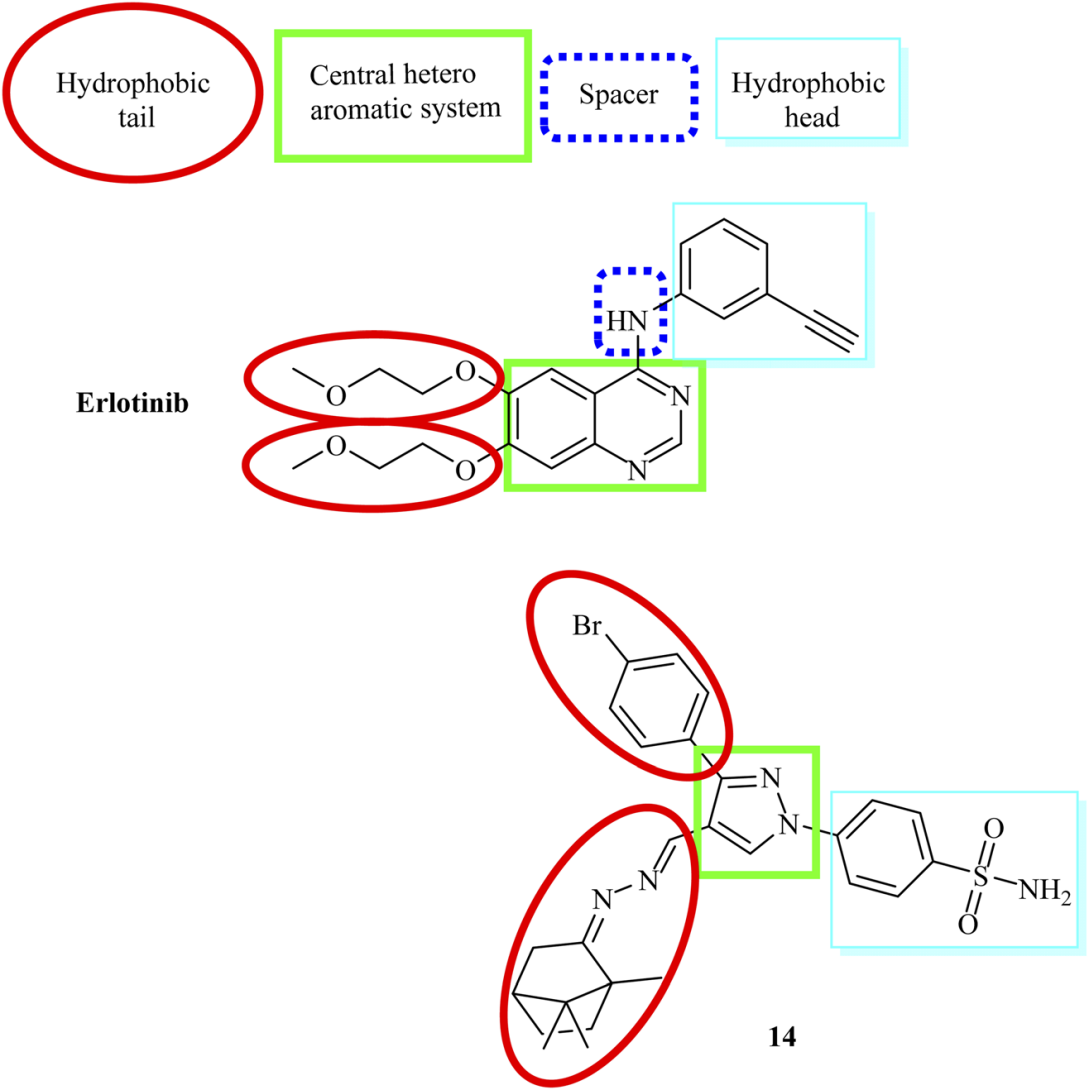
Essential necessities for EGFR inhibitors.

Our structures exhibited the necessary requirements of EGFR inhibitors (Fig. 2 and 3). The bio-isosteric replacements were at four points of erlotinib as EGFR inhibitor: (1) a hydrophobic tail (occupy hydrophobic region II), (2) HBD NH group and/or HBA, (3) a hydrophobic domain (occupy hydrophobic area I), and (4) a flat heteroaromatic ring which inhabits the adenine site (Fig. 2 and 3)^62–64^

## 3. Results and discussion

### 3.1. Chemistry

**Scheme 1:**
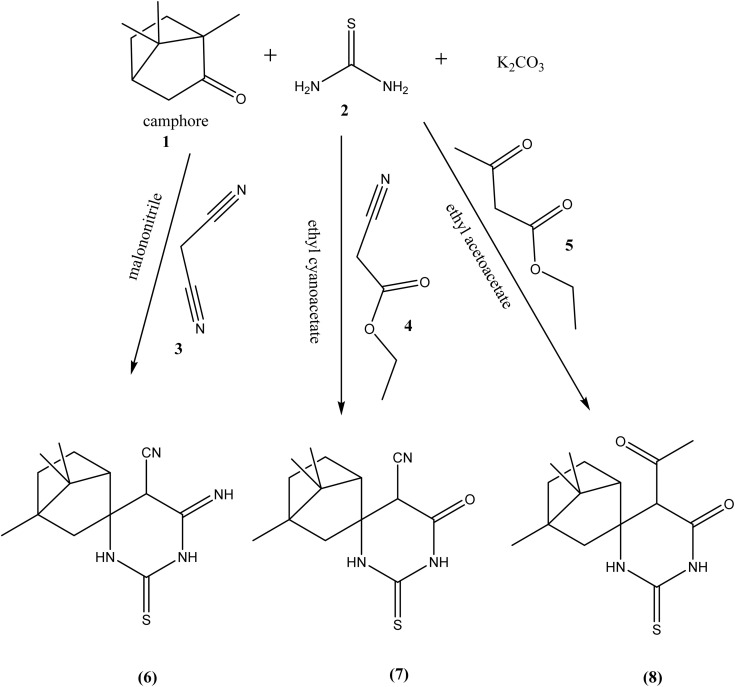
Synthesis of compounds 6–8.

**Scheme 2:**
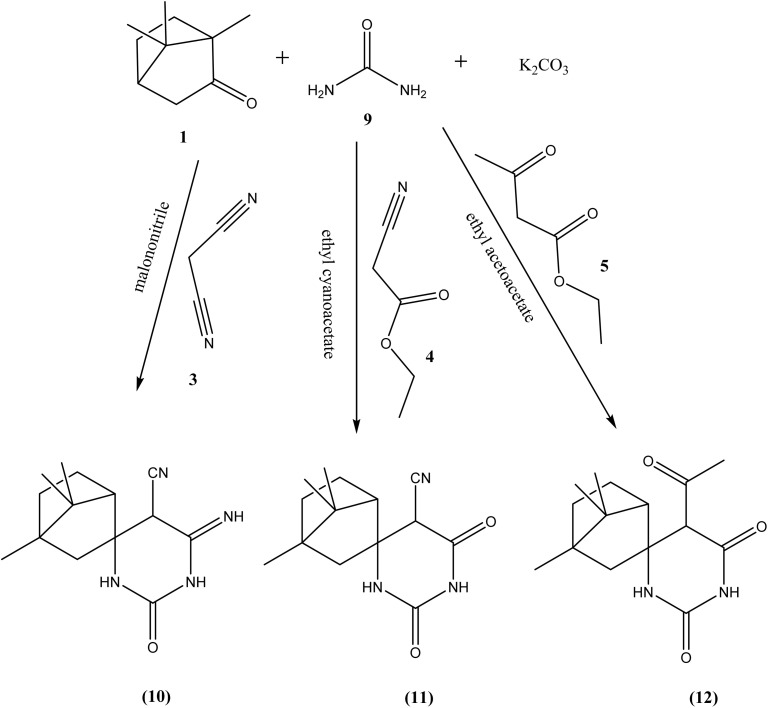
Synthesis of compounds 10–12.

**Scheme 3:**
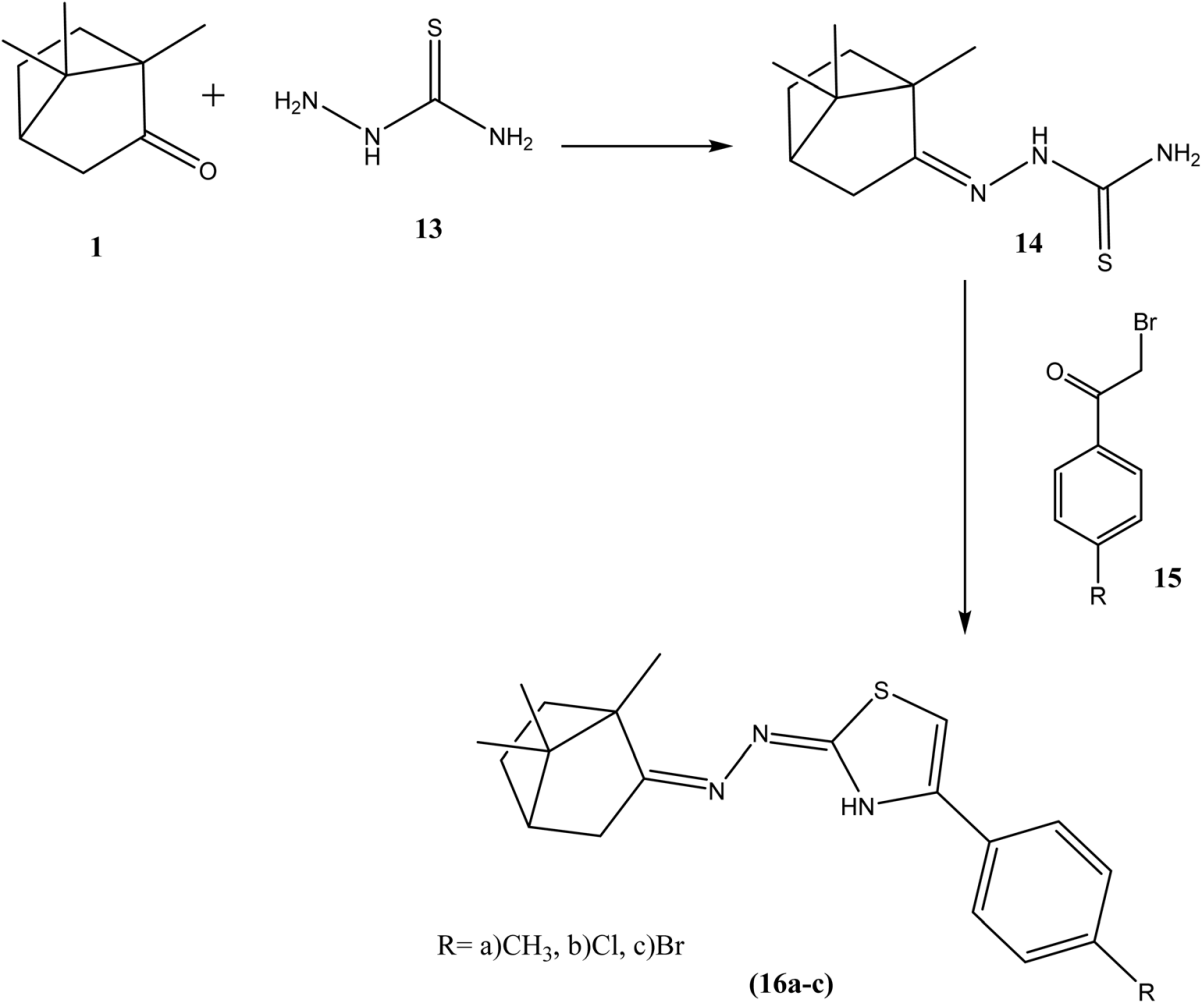
Synthesis of compounds 16a–c.

**Scheme 4:**
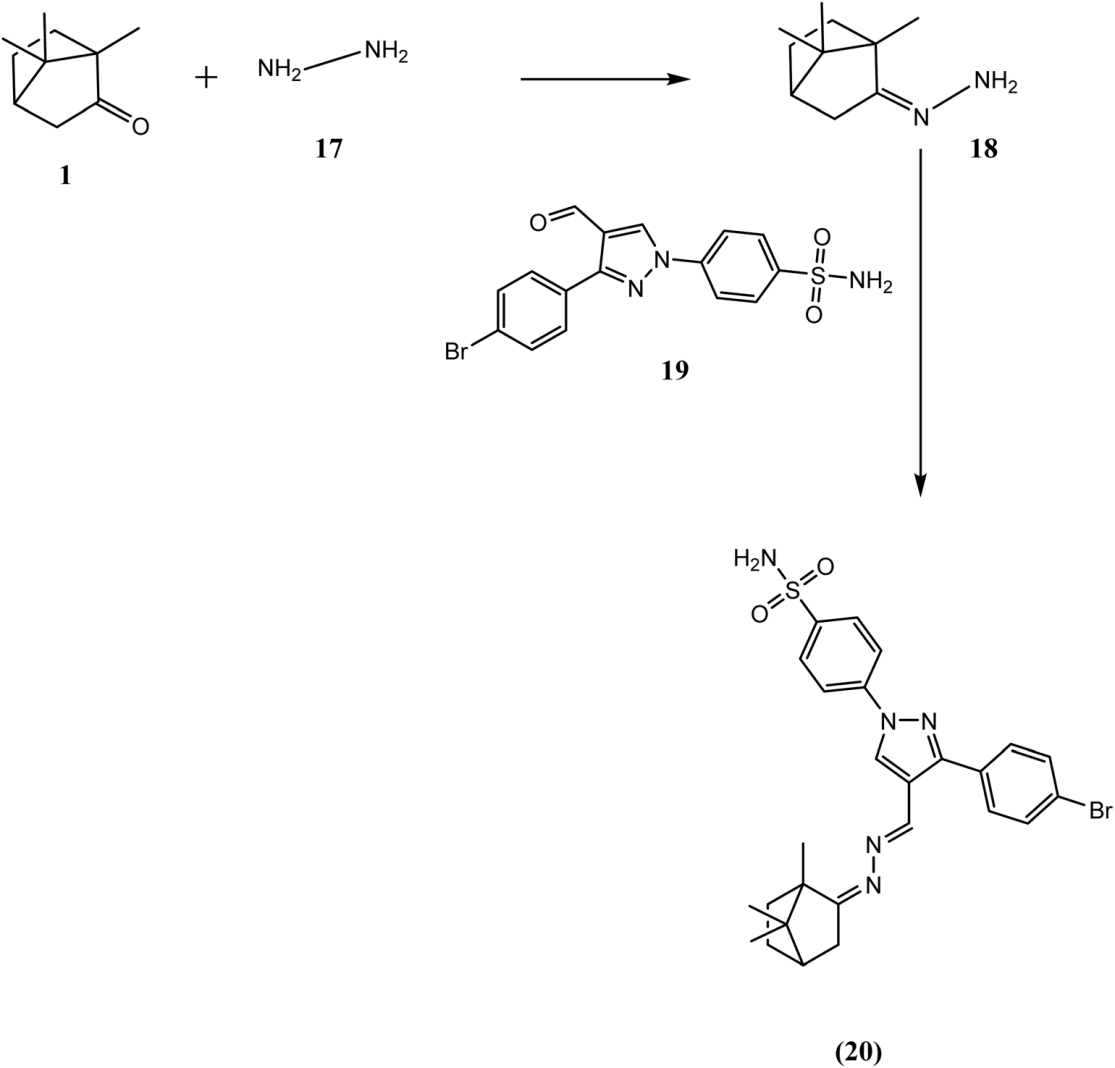
Synthesis of compound 20.

The synthetic pathway for derivatives 6–20 is illustrated in Scheme 1–4. Starting with a mixture of camphor 1 with thiourea 2 in the presence of potassium carbonate which heated under reflux with the appropriate malononitrile, ethyl cyanoacetate and/or ethyl acetoacetate to give the corresponding derivatives 6, 7 and 8 respectively (Scheme 1). Moreover, cyclocondensation of the same components but using urea instead of thiourea resulted in the corresponding derivatives 10, 11 and 12 respectively (Scheme 2). In addition, condensation of camphor with thiosemicarbazide 13 yielded the corresponding thiosemicarbazone 14 that underwent cyclocondensation with 2-bromo-1-(substituted) ethan-1-one to give the consistent derivatives 16a–c correspondingly (Scheme 3). Finally, the condensation reaction of camphor with hydrazine hydrate 17 produced the corresponding Schiff's base 18 which also condensed with the aldehyde derivative 19 to afford the corresponding derivative 20 respectively (Scheme 4).

### 3.2. Biological activities

**Fig. 4:**
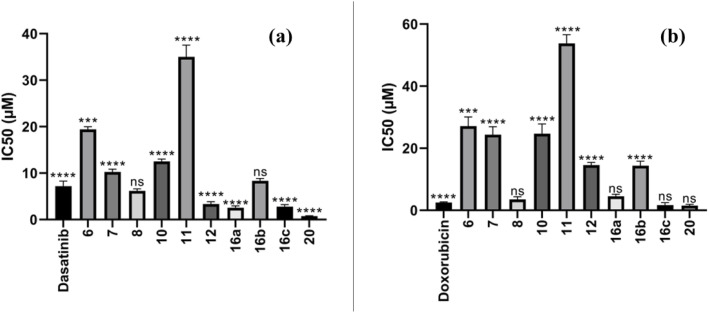
IC_50_ of the synthesized compounds (a) against of MCF-7 and (b) against of A549 cell lines and reference drugs dasatinib, erlotinib & doxorubicin. Values are mean ± SD. Asterisks indicate significant differences from the respective control (dasatinib for MCF-7 and doxorubicin for A549) as revealed by one-way ANOVA test, *P < 0.05, **P < 0.01, ***P < 0.001 and ****P < 0.0001.

MCF-7 Adenocarcinoma of the breast in humans and A549 small lung cancer lines of cells (purchased from Labco's Sigma-Aldrich) were employed to evaluate the newly synthesized compounds' anti-cancer potential. The IC_50_ values of these compounds were assessed using the MTT test for cell viability as shown in Table 1. The compounds demonstrated significant inhibitory activity against MCF-7, with IC_50_ values of 0.78 and 19.92 μM, compared to dasatinib (IC_50_ = 7.99 μM), erlotinib (IC_50_ = 13.91 μM) and doxorubicin (IC_50_ = 3.10 μM) as shown in Fig. 4a. Notably, compound 20 exhibited excellent inhibitory activity (IC_50_ = 0.78 μM), proving to be more potent than standard drugs, dasatinib (IC_50_ = 7.99 μM), erlotinib (IC_50_ = 13.91 μM) and doxorubicin (IC_50_ = 3.10 μM). In contrast, the compounds showed moderate inhibitory effects against A549 cells, with IC_50_ values ranging from 1.69 to 56.6 μM, compared to dasatinib (IC_50_ = 11.8 μM), erlotinib (IC_50_ = 5.49 μM) and doxorubicin (IC_50_ = 2.43 μM). Similarly, compound 20 displayed strong inhibitory activity (IC_50_ = 1.69 μM), again surpassing the standard drugs, dasatinib (IC_50_ = 11.8 μM), erlotinib (IC_50_ = 5.49 μM) and doxorubicin (IC_50_ = 2.43 μM) as shown in Fig. 4.

**Table 1:** *In vitro* MCF-7 and A549 inhibitory activity of the synthesized compounds against of MCF-7 and A549 cell lines and reference drugs dasatinib, erlotinib & doxorubicin. Values are mean ± SD. Asterisks indicate significant differences from the respective control (Dasatinib for MCF-7 and Doxorubicin for A549) as revealed by one-way ANOVA test, *P < 0.05, **P < 0.01, ***P < 0.001 and ****P < 0.0001

Compound	Cytotoxicity IC_50_ (μM)^a^	
	MCF-7	A549
6	19.92 ± 0.73^***^	28.47 ± 0.99^****^
7	10.11 ± 0.39^****^	22.24 ± 0.77^****^
8	6.15 ± 0.23	3.34 ± 0.12
10	12.66 ± 0.47^****^	23.27 ± 0.81^****^
11	35 ± 1.32^****^	56.6 ± 2.01^****^
12	3.20 ± 0.12^****^	14.38 ± 0.5^****^
16a	2.16 ± 0.08^****^	4.13 ± 0.14
16b	8.29 ± 0.3	15.25 ± 0.53^****^
16c	2.84 ± 0.1^****^	1.29 ± 0.04
20	0.78 ± 0.03^****^	1.69 ± 0.06
Dasatinib	7.99 ± 0.29	11.81 ± 0.41
Erlotinib	13.91 ± 1.3	5.49 ± 0.45
Doxorubicin	3.12 ± 0.11	2.43 ± 0.08

a IC_50_ values are the mean ± SD of three experiments separately.

### 3.3. EGFR kinase inhibitory activity

**Fig. 5:**
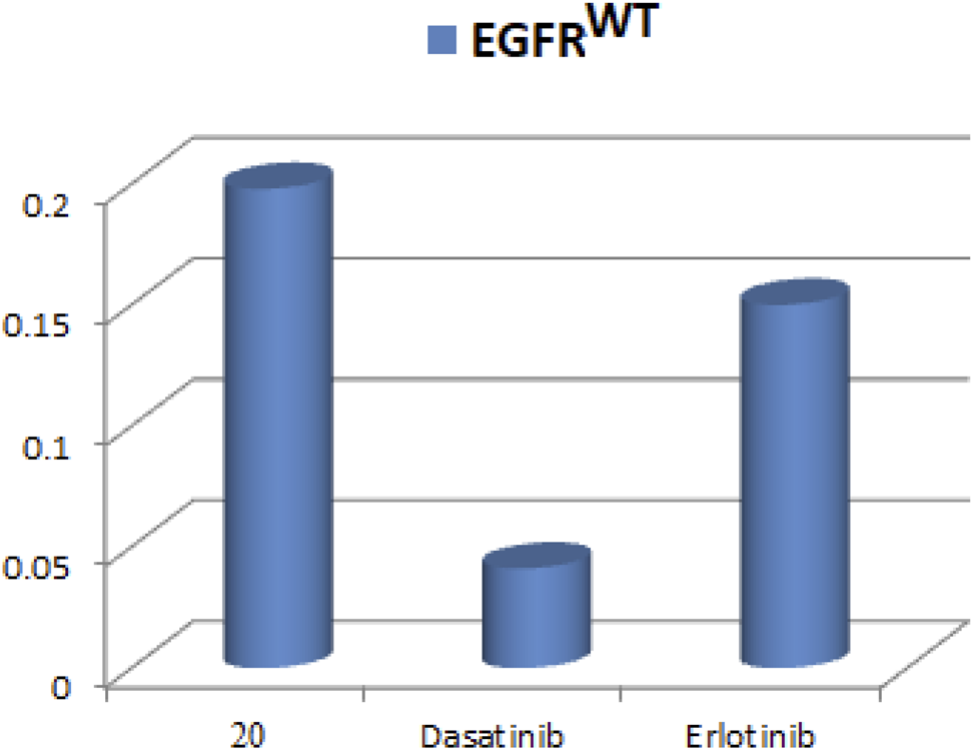
IC_50_ values of compound (20) compared to dasatinib on EGFR.

The EGFR (Catalog # 40321) Kinase Assay Kit was utilized to assess the EGFR kinase inhibitory activity of compound 20 as shown in Fig. 5. Five different concentrations were evaluated in this assay, with erlotinib and dasatinib serving as reference drugs for determining the IC_50_. The results indicated that compound 20 exhibited enhanced inhibitory activity against EGFR^WT^, with an IC_50_ of 0.198 μM, which is comparable to erlotinib and dasatinib's IC_50_ of 0.15 and 0.041 μM respectively.

### 3.4. Molecular docking

For docking studies, Molsoft program was used. The ID of the PDB for EGFR^WT^ wild-type (PDB ID 4HJO) ^65^ was used.

**Fig. 6:**
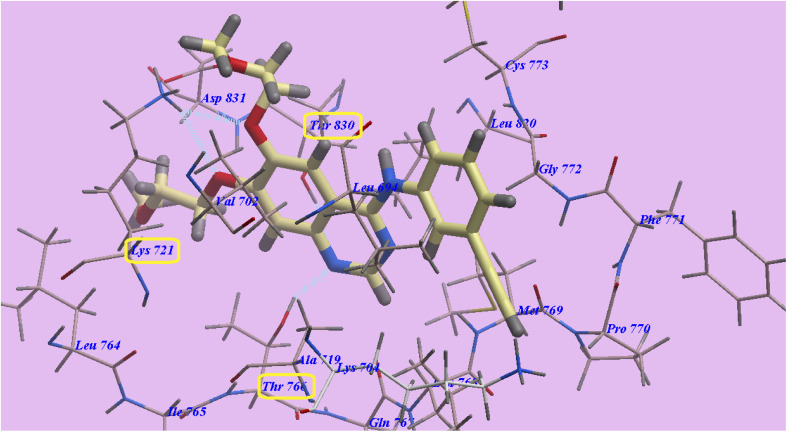
Docking of erlotinib in 4HJO active site.

Erlotinib in its normal form shows binding energy = −92.64 kcal mol^−1^. Two hydrogen bonds were formed with Cys773 (2.95 Å) and Met769 (2.00 Å). The 3-ethynylphenyl head occupied the hydrophobic region I, which was produced by *Val702*, *Asp831*, *Lys721*, *Ile765*, *Thr766*, *Ala719*, and *Thr830*. Additionally, the hydrophobic furrow II formed by *Leu694*, *Asp776*, *Cys773*, *Leu820*, *Gly772*, *Phe771*, *Met769*, and *Pro770* were occupied by the 2-methoxyethoxy side chains as shown in (Fig. 6).

**Fig. 7:**
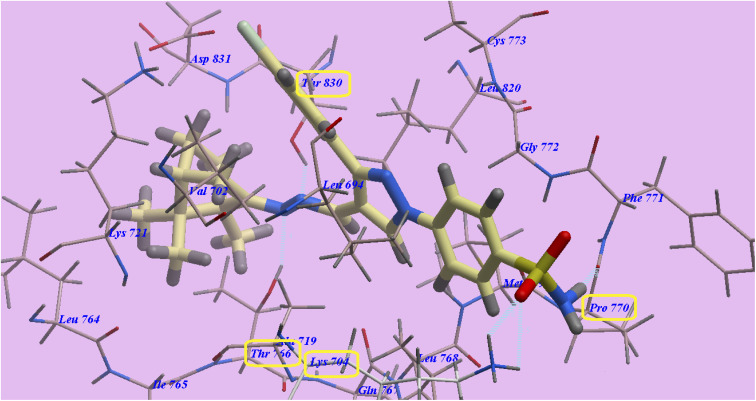
Docking of compound 20 in 4HJO.

Compound 20 exhibited nearly the same pattern as that of erlotinib with affinity of −150.65 kcal mol^−1^ and made 6 H-bonds with *Asp837* (2.08 Å), *Thr854* (2.97 Å), *Met793* (1.74 Å), *Gln791* (2.56 Å), *Glu762* (2.22 Å) and *Lys875* (2.06 Å) (Fig. 7).

### 3.5. Validation of the accuracy of docking

**Fig. 8:**
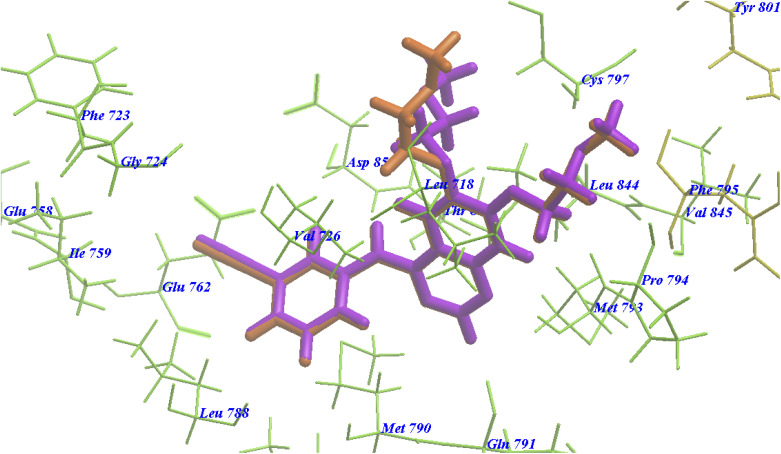
Superimposition of erlotinib on the co-crystallized native bound one with 4HJO.

The re-docked erlotinib seems exactly superimposed on the co-crystallized native bound one (Fig. 8). These results indicated the high accuracy of the docking simulation in comparison with the biological methods.

### 3.6. Pharmacokinetics and ADME analysis

In drug development, bioavailability is a factor that must be considered in clinical applications. It is affected by the different physicochemical properties of compounds.^66^

The most active compounds and the standard dasatinib and Erlotinib were subjected to view physicochemical properties and drug likeness using the SwissADME site free web tool, developed by the Molecular Modelling Group of the Swiss Institute of Bioinformatics.

In particular, the compliance of compounds to traditional Lipinski's “rule of five” was calculated to determine if the compound can be orally active in human.^67^ This simple rule states that orally active drug has no more than one violation of the following criteria: molecular weight (MW) less than 500 Da; no more than 10 hydrogen bond acceptors (HBA); no more than five hydrogen bond donors (HBD); and calculated octanol–water partition coefficient (*i* log *P*) not greater than 573. Also, the topological polar surface area (TPSA) and the number of rotatable bonds is other critical properties that have been linked to drug bioavailability.^68^ The reports suggested that compounds with a TPSA of more than 140 Å^2^ and more than 10 rotatable bonds are thought to have low pharmacological flexibility and permeability, respectively.^69^ All of the tested compounds have a number of hydrogen bond donor atoms of 1–3 and hydrogen bond acceptor atoms of 2–7 compared to 3 hydrogen bond donors and 7 hydrogen bond acceptors in the standard dasatinib. The number of rotatable bonds of the tested compounds ranged from 0–5 bonds, while dasatinib contains 8 bonds. The molar refractivity (MR) value should be between 40 and 130 for good absorption and oral bioavailability. Acceptable molar refractivity values, in combination with the number of rotatable bonds, indicate that substances have adequate intestinal absorption and oral bioavailability.^69^ The designed compound's MR values range from 76.56 to 143.35 m^3^ mol^−1^, compared to the standard dasatinib (MR 133.62 m^3^ mol^−1^). This indicates that most of the proposed compounds (except compound 6 and the standard dasatinib) may have low gastrointestinal absorption and oral bioavailability. Also, the proposed derivatives' TPSA values were range from 68.75 Å^2^ to 111.08 Å^2^ compared to TPSA value of dasatinib 134.75 Å^2^. However, compounds showed TPSA values lower than the standard range. It is worth mentioning that compounds with TPSA values lower than 60 Å2 can penetrate the BBB easily.^69^ That's why all the proposed derivatives and the standard dasatinib cannot penetrate the blood–brain barrier well, as evidenced by the BBB determination. In addition, the implicit log *P* (*I* log *P*) *n*-octanol/water partition coefficients were known to play a critical role in medication absorption in the mouth, as well as facilitating drug interactions with their biological targets.^67^ The estimated values of *I* log *P* were found to be less than five (1.47–4.00), as recommended by Lipinski's rule of five and compared to dasatinib (3.15). Moreover, the estimated aqueous solubility of the tested compounds Log S (ESOL) ranged from −2.51 to −50.90 compared to −4.84 of dasatinb, indicating their low aqueous solubility (soluble compounds have log *S* < −4). Furthermore, studying the inhibitory activity of proposed derivatives against a certain CYP isoform becomes a critical factor.^67^ While all of the target compounds and dasatinib were found not to inhibit CYP1A2. The derivatives 16b, 16c, 20 and the standard dasatinib were anticipated to inhibit CYP2C19 and The derivatives 7, 8, 16b, 16c, 20 and the standard dasatinib were anticipated to inhibit CYP2C9 enzymes which means they are lower exposed to inhibitory drug metabolism.

## 4. Materials and methods

### 4.1. Chemistry

#### 4.1.1. General

We used an uncorrected Thomas-Hoover capillary equipment to find the melting points. A Nicolet 550 Series II Magna FT-IR spectrometer was used to acquire infrared (IR) spectra as films on KBr plates. Beni-Suef University in Egypt's Faculty of Pharmacy used a Bruker 400 MHz NMR spectrometer to record nuclear magnetic resonance (NMR) spectra. Tetramethyl silane (TMS) served as the internal standard, and the solvents utilized were CDCl3 or DMSO-d6. The estimated *J* values, or coupling constants, were expressed in hertz (Hz).

### 4.2. Experimental procedures and spectral data for compounds (6–12)

A reaction mixture consisting of camphor (0.1 mole), potassium bicarbonate (0.01 mole), the relevant malononitrile, ethyl cyanoacetate and/or ethyl acetoacetate (0.01 mole), and thiourea and/or urea (0.01 mole) was prepared in 30 ml of absolute ethanol and subjected to reflux for 4 hours. The resulting precipitate was collected by filtration and subsequently dried upon cooling. Physical and spectral data for them are listed below.

#### 4.2.1. 6′-Imino-4,7,7-trimethyl-2′-thioxotetrahydro-1′*H*-spiro[bicyclo[2.2.1]heptane-2,4′-pyrimidine]-5′-carbonitrile (6)

Dark green solid, yield (80%), m.p.: 220–223 °C (ethanol), IR (KBr, cm^−1^) *ν* = 3327 (NH), 3175 (C–H aromatic), 2977 (C–H aliphatic), 1630 (C=N), 1540 (CN), 1114 (C=S), 611 (S); ^1^H NMR (DMSO-*d*_6_) at *δ* = 0.9–1.5 (s, 9H, 3CH_3_), 1.6–2.4 (m, 6H, aliphatic protons), 7.45 (s, 1H, NH), 9.2 (s, 1H, C=NH), 10.32 (s, 1H, NH), ^13^C-NMR (100 MHz) (DMSO-*d*_6_) at *δ* = 18.02, 18.23, 18.96, 21.43, 31.9, 35.2, 37.08, 39.9, 41.12, 41,45, 47.33, 118.22, 156.3, 182.1 anal. calcd. For C_14_H_20_N_4_S (276.14): C, 60.84; H, 7.29; N, 20.27; S, 11.60. Found C = 60.85; H = 7.30; N, 20.25; S = 11.62.

#### 4.2.2. 4,7,7-Trimethyl-6′-oxo-2′-thioxotetrahydro-1′*H*-spiro[bicyclo[2.2.1]heptane-2,4′-pyrimidine]-5′-carbonitrile (7)

Buff solid, yield 89%, m.p.203–205 °C (ethanol), IR (KBr) 3436 (C–H aromatic), 1619 (C=O), 1401 (CN), 1114 (C=S), 612 (S) cm^−1^); ^1^H NMR (DMSO-*d*_6_) at *δ* = 0.9–1.5 (s, 9H, CH_3_), 1.6–2.4 (m, 6H, aliphatic protons), 7.45 (s, 1H, NH), 9.2 (s, 1H, C=NH), 10.32 (s, 1H, NH), ^13^C-NMR (100 MHz) (DMSO-*d*_6_) at *δ* = 18.02, 18.23, 18.96, 21.43, 31.9, 35.2, 37.08, 39.9, 41.12, 41,45, 47.33, 118.22, 156.3, 182.1 anal. calcd. For C_14_H_19_N_3_OS (277.12): C, 60.62; H, 6.90; N, 15.15; O, 5.77; S, 11.56. Found C = 60.62; H = 6.90; N= 15.15; O = 5.77; *S* = 11.56.

#### 4.2.3. 5′-Acetyl-4,7,7-trimethyl-2′-thioxodihydro-1′*H*-spiro[bicyclo[2.2.1]heptane-2,4′-pyrimidin]-6′(5′*H*)-one (8)

Off-white solid, yield 70%, m.p.213–215 °C (ethanol), IR (KBr) 3334 (NH), 3197 (C–H aromatic), 1640 (C=O), 1141 (C=S), 610 (S) cm^−1^); ^1^H NMR (DMSO-*d*_6_) at *δ* = 0.9–1.5 (s, 9H, CH_3_), 1.6–2.4 (m, 6H, aliphatic protons), 7.45 (s, 1H, NH), 9.2 (s, 1H, C=NH), 10.32 (s, 1H, NH), ^13^C-NMR (100 MHz) (DMSO-*d*_6_) at *δ* = 18.02, 18.23, 18.96, 21.43, 31.9, 35.2, 37.08, 39.9, 41.12, 41,45, 47.33, 118.22, 156.3, 182.1 anal. calcd. For C_15_H_22_N_2_OS (278.15): C, 64.71; H, 7.96; N, 10.06; O, 5.75; S, 11.52. Found C = 64.71; H = 7.96; N= 10.06; O = 5.75; *S* = 11.52.

#### 4.2.4. 6′-Imino-4,7-trimethyl-2′-oxotetrahydro-1′*H*-spiro[bicyclo[2.2.1]heptane-2,4′-pyrimidine]-5′-carbonitrile (10)

Dark green solid, yield 82%, m.p.209–212 °C (ethanol), IR (KBr) 3412 (NH), 3180 (C–H aromatic), 1643 (C=O), 1402 (CN), 1114 (C=O) cm^−1^); ^1^H NMR (DMSO-*d*_6_) at *δ* = 0.9–1.5 (s, 9H, CH_3_), 1.6–2.4 (m, 6H, aliphatic protons), 7.45 (s, 1H, NH), 9.2 (s, 1H, C=NH), 10.32 (s, 1H, NH), ^13^C-NMR (100 MHz) (DMSO-*d*_6_) at *δ* = 18.02, 18.23, 18.96, 21.43, 31.9, 35.2, 37.08, 39.9, 41.12, 41,45, 47.33, 118.22, 156.3, 182.1 anal. calcd. For C_15_H_20_N_4_O (272.16): C, 66.15; H, 7.40; N, 20.57; O, 5.87. Found C = 66.15; H = 7.40; N= 20.57; O = 5.87.

#### 4.2.5. 4,7,7-Trimethyl-2′,6′-dioxotetrahydro-1′*H*-spiro[bicyclo[2.2.1]heptane-2,4′-pyrimidine]-5′-carbonitrile (11)

Buff solid, yield 69%, m.p.205–207 °C (ethanol), IR (KBr) 3464 (NH), 3175 (C–H aromatic), 2981 (C–H aliphatic), 1613 (C=O), 1362 (CN) cm^−1^); ^1^H NMR (DMSO-*d*_6_) at *δ* = 0.9–1.5 (s, 9H, CH_3_), 1.6–2.4 (m, 6H, aliphatic protons), 7.45 (s, 1H, NH), 9.2 (s, 1H, C=NH), 10.32 (s, 1H, NH), ^13^C-NMR (100 MHz) (DMSO-*d*_6_) at *δ* = 18.02, 18.23, 18.96, 21.43, 31.9, 35.2, 37.08, 39.9, 41.12, 41,45, 47.33, 118.22, 156.3, 182.1 anal. calcd. For C_14_H_19_N_3_O_2_ (261.15): C, 64.35; H, 7.33; N, 16.08; O, 12.25. Found C = 64.35; H = 7.33; N= 16.08; O = 12.25.

#### 4.2.6. 5′-Acetyl-4,7,7-trimethyl-1′*H*-spiro[bicyclo[2.2.1]heptane-2,4′-pyrimidine]-2′,6′(3′*H*,5′*H*)-dione (12)

Off-white solid, yield 57%, m.p.200–203 °C (ethanol), IR (KBr) 3393 (NH), 3136 (C–H aromatic), 2954 (C–H aliphatic), 1632 (C=O) cm^−1^); ^1^H NMR (DMSO-*d*_6_) at *δ* = 0.9–1.5 (s, 9H, CH_3_), 1.6–2.4 (m, 6H, aliphatic protons), 7.45 (s, 1H, NH), 9.2 (s, 1H, C=NH), 10.32 (s, 1H, NH), ^13^C-NMR (100 MHz) (DMSO-*d*_6_) at *δ* = 18.02, 18.23, 18.96, 21.43, 31.9, 35.2, 37.08, 39.9, 41.12, 41,45, 47.33, 118.22, 156.3, 182.1 anal. calcd. For C_15_H_22_N_2_O_3_ (278.16): C, 64.73; H, 7.97; N, 10.06; O, 17.24. Found C = 64.73; H = 7.97; N= 10.06; O = 17.24.

### 4.3. Experimental procedures and spectral data for compounds (16a–c)

Camphor-thiosemicarbazone was synthesized following the method described by Brousse BN *et al.*,^70^ This process entails the reaction of camphor with thiosemicarbazide in heated ethanol, accompanied by a few drops of sulfuric acid. A mixture of camphor-thiosemicarbazone (0.1 mole) and phenacyl bromide derivatives (0.1 mole) in 30 ml of absolute ethanol, in the presence of sodium acetate, was refluxed for 24 hours. The resulting precipitate was collected by filtration and dried after cooling. Physical and spectral data for them are listed below.

#### 4.3.1. 4-(*p*-Tolyl)-2-1,7,7-trimethylbicyclo[2.2.1]heptan-2-ylidene)hydrazineylidene)-2,3-dihydrothiazole (16a)

Black solid, yield 60%, m.p.213–215 °C (ethanol), IR (KBr) 3425 (NH), 3155 (C–H aromatic), 2955 (C–H aliphatic), 1600 (C=C), 1404 (C=N), 614 (S) cm^−1^); ^1^H NMR (DMSO-*d*_6_) at *δ* = 0.9–1.5 (s, 9H, CH_3_), 1.6–2.4 (m, 6H, aliphatic protons), 7.45 (s, 1H, NH), 9.2 (s, 1H, C=NH), 10.32 (s, 1H, NH), ^13^C-NMR (100 MHz) (DMSO-*d*_6_) at *δ* = 18.02, 18.23, 18.96, 21.43, 31.9, 35.2, 37.08, 39.9, 41.12, 41,45, 47.33, 118.22, 156.3, 182.1 anal. calcd. For C_20_H_25_N_3_S (339.18): C, 70.76; H, 7.42; N, 12.38; S, 9.44. Found C = 70.76; H = 7.42; N= 12.38; *S* = 9.44.

#### 4.3.2. 4-(4-Chlorophenyl)-2-1,7,7-trimethylbicyclo[2.2.1]heptan-2-ylidene)hydrazineylidene)-2,3-dihydrothiazole (16b)

Brown solid, yield 64%, m.p.223–225 °C (ethanol), IR (KBr) 3430 (NH), 2959 (C–H aliphatic), 1625 (C=C), 1560 (C=N), 613 (Br) cm^−1^); ^1^H NMR (DMSO-*d*_6_) at *δ* = 0.9–1.5 (s, 9H, CH_3_), 1.6–2.4 (m, 6H, aliphatic protons), 7.45 (s, 1H, NH), 9.2 (s, 1H, C=NH), 10.32 (s, 1H, NH), ^13^C-NMR (100 MHz) (DMSO-*d*_6_) at *δ* = 18.02, 18.23, 18.96, 21.43, 31.9, 35.2, 37.08, 39.9, 41.12, 41,45, 47.33, 118.22, 156.3, 182.1 anal. calcd. For C_19_H_22_ClN_3_S (359.12): C, 63.40; H, 6.16; Cl, 9.85; N, 11.67; S, 8.91. Found C = 63.40; H = 6.16; Cl = 9.85; N= 11.67; S = 8.91.

#### 4.3.3. 4-(4-Bromophenyl)-2-1,7,7-trimethylbicyclo[2.2.1]heptan-2-ylidene)hydrazineylidene)-2,3-dihydrothiazole (16c)

Dark green solid, yield 68%, m.p.234–236 °C (ethanol), IR (KBr) 3422 (NH), 3161 (C–H aromatic), 2956 (C–H aliphatic), 1560 (C=N), 1540 (CN), 613 (Cl) cm^−1^); ^1^H NMR (DMSO-*d*_6_) at *δ* = 0.9–1.5 (s, 9H, CH_3_), 1.6–2.4 (m, 6H, aliphatic protons), 7.45 (s, 1H, NH), 9.2 (s, 1H, C=NH), 10.32 (s, 1H, NH), ^13^C-NMR (100 MHz) (DMSO-*d*_6_) at *δ* = 18.02, 18.23, 18.96, 21.43, 31.9, 35.2, 37.08, 39.9, 41.12, 41,45, 47.33, 118.22, 156.3, 182.1 anal. calcd. For C_19_H_22_BrN_3_S (403.07): C, 56.43; H, 5.48; Br, 19.76; N, 10.39; S, 7.93 Found C = 56.43; H = 5.48; Br = 19.76; *N* = 10.39; S = 7.93.

### 4.4. Experimental procedures and spectral data for compound (20)

Camphor (0.1 mole) and hydrazine hydrate (0.1 mole) were dissolved in 20 ml of ethanol and transferred to a round-bottom flask. The reaction mixture was acidified with glacial acetic acid and subsequently refluxed in a water bath for 5 hours. Upon cooling, the residue was isolated, filtered, dried, and recrystallized from a 1 : 1 mixture of benzene and hexane, yielding a colorless crystalline powder.^71^ A mixture of camphor-hydrazone (0.1 mole) and brominated aldehyde (0.1 mole) in 30 ml of absolute ethanol was refluxed for 20 hours. The precipitate formed upon cooling was then filtered and dried at room temperature, resulting in the production of compound 20. Physical and spectral data for them are listed below.

#### 4.4.1. 4-(3-(4-Bromophenyl)-4-1,7,7-trimethylbicyclo[2.2.1]heptan-2-ylidene)hydrazineylidene)-methyl)-1*H*-pyrazol-1-yl)benzenesulfonamide (20)

Pale green solid, yield 90%, m.p. 283–285 °C (ethanol), IR (KBr) 3434 (NH), 3175 (C–H aromatic), 2977 (C–H aliphatic), 1632 (C=O), 1402 (C=N), 1129 (C–N), 616 (Br) cm^−1^); ^1^H NMR (DMSO-*d*_6_) at *δ* = 0.9–1.5 (s, 9H, CH_3_), 1.6–2.4 (m, 6H, aliphatic protons), 7.45 (s, 1H, NH), 9.2 (s, 1H, C=NH), 10.32 (s, 1H, NH), ^13^C-NMR (100 MHz) (DMSO-*d*_6_) at *δ* = 18.02, 18.23, 18.96, 21.43, 31.9, 35.2, 37.08, 39.9, 41.12, 41,45, 47.33, 118.22, 156.3, 182.1 anal. calcd. For C_26_H_28_BrN_5_O_2_S (553.11): C, 56.32; H, 5.09; Br, 14.41; N, 12.63; O, 5.77; S, 5.78. Found C = 56.32; H = 5.09; Br = 14.41; N= 12.63; O = 5.77; S = 5.78.

### 4.5. Biological activities

#### 4.5.1. Cell culture protocol

The American type culture collection provided the cell lines and were cultured in 10% FBS added to DMEM (Invitrogen/Life Technologies) (Hyclone), a 10 μg ml^−1^ of insulin (Sigma), and a 1% penicillin-streptomycin. The suppliers of all other chemicals and reagents were either Invitrogen or Sigma.

For the MTT assay, cells were plated at a density of 1.2 to 1.8 × 10 000 cells per well in a total volume of 200 μl, comprising 100 μl of a full growing medium and 100 μl of the substance that was evaluated in each well. The cells were then incubated for 24 hours.

### 4.6. Cell culture steps

The culture medium should be transferred to a centrifuge tube. To get rid of serum, quickly rinse the cell layer with a solution of 0.53 mM EDTA and 0.25% (w/v) trypsin, which contains trypsin inhibitors. When the cell layer is fully scattered, which usually happens in 5 to 15 minutes, look at the cells under an inverted microscope after adding 2.0 to 3.0 ml of the Trypsin–EDTA solution to the flask.

Note: To prevent clumping, avoid agitating the flask while the cells detach. If detachment proves difficult, incubate the flask at 37 °C to facilitate the process.

Aspirate the cells gently using a pipette after adding 6.0 to 8.0 ml of full growth media. Put the cell suspension and the first step's medium in the centrifuge tube, then centrifuge for five to ten minutes at about 125 × *g*. Transfer the proper aliquots to new culture vessels, discard the supernatant, and resuspend the cell pellet in fresh growth media. For a full day, incubate the cultures at 37 °C. After the chemical has been treated with repeated concentrations, incubate for a further 48 hours at 37 °C. To perform the MTT assay, examine the plates using an inverted microscope.

### 4.7. MTT – cytotoxicity assay protocol

The MTT method is highly effective for evaluating *in vitro* cytotoxicity in multi-well plates. For best results, utilize cells in the logarithmic phase of growth, ensuring that the final cell density does not surpass 10^6^ cells per cm^2^. Additionally, incorporate a blank control containing complete medium without any cells. Remove the cultures from the incubator and transfer them to a laminar flow hood or sterile workspace. Reconstitute each vial of MTT [M-5655] with 3 ml of medium or balanced salt solution that is free from phenol red and serum. Add the reconstituted MTT in an amount equal to 10% of the total volume of the culture medium. Return the cultures to the incubator for 2 to 4 hours, depending on the cell type and maximum cell density; typically, 2 hours is adequate, though longer incubation may be required for low-density or less metabolically active cells. It is important to maintain consistent incubation times for accurate comparisons.

After incubation, remove the cultures from the incubator and dissolve the formed formazan crystals by adding an amount of MTT solution [M-8910] equivalent to the original culture medium volume. Gentle mixing with a gyratory shaker will facilitate the dissolution of the crystals. In denser cultures, pipetting up and down may be necessary to ensure complete dissolution of the MTT formazan crystals. Measure the absorbance spectrophotometrically at 570 nm and assess the background absorbance of the multi-well plates at 690 nm, which should be subtracted from the 570 nm measurement. The tests in multi-well plates can be read using an appropriate plate reader, or the contents can be transferred to cuvettes for measurement.

### 4.8. EGFR kinase inhibitory activity protocol

The EGFR (Catalog # 40321) Kinase Assay Kit was employed to assess the EGFR kinase inhibitory activity of compound 20. Begin by thawing ATP, Poly (Glu : Tyr 4 : 1), and PTK substrate (10 mg ml^−1^) in 5× Kinase Buffer 1. Optionally, to achieve a 10 mM concentration, add DTT to the 5× Kinase Buffer 1 (for example, by adding 10 μl of 1 M DTT to 1 ml of 5× Kinase Buffer 1).

Prepare the primary mixture (25 μl per well) by combining 6 μl of 5× Kinase Buffer 1, 1 μl of ATP (500 μM), 1 μl of PTK substrate Poly (Glu : Tyr 4 : 1) (10 mg ml^−1^), and 17 μl of water. Distribute this mixture into N wells, adding 25 μl to each well. Then, introduce 5 μl of the “Test Inhibitor” solution into the appropriate well. For the “Positive Control” and “Blank” samples, add 5 μl of the same solution without the inhibitor (Inhibitor buffer).

To prepare 3 ml of 1× Kinase Buffer 1, combine 2400 μl of water with 600 μl of 5× Kinase Buffer 1. This 3 ml of 1× Kinase Buffer 1 is sufficient for 100 reactions. For the “Blank” wells, add 20 μl of 1× Kinase Buffer 1. Thaw the EGFR enzyme on ice, and once the tube is fully defrosted, briefly spin it to collect all contents. Determine the required amount of EGFR for the assay, then dilute the enzyme in 1× Kinase Buffer 1 to achieve a concentration of 1 ng μl^−1^. Any remaining undiluted enzyme should be aliquoted and stored at −80 °C. Note that the EGFR enzyme is sensitive to freeze-thaw cycles, so these should be minimized. Reusing thawed aliquots or diluted enzymes is not recommended.

To initiate the reaction, add 20 μl of the diluted EGFR enzyme to both the “Positive Control” and “Test Inhibitor Control” wells. Incubate the mixture for 40 minutes at 30 °C. After this incubation, thaw the Kinase-Glo Max solution. Following the 40-minutes reaction, add 50 μl of the Kinase-Glo Max reagent to each well. Cover the plate with aluminum foil and incubate for an additional 15 minutes at room temperature. Finally, measure luminescence using a microplate reader.

### 4.9. Molecular docking

Molsoft program was used for docking studies. The protein of EGFR^WT^ wild-type (PDB ID 4HJO) ^65^ was used. The protein was obtained from the Brookhaven Protein Databank. It was prepared for docking by adding polar hydrogens to its atoms. The protein binding site was defined by overlaying a grid on the center of the co-crystallized ligand. Before conducting docking simulations, all requisite grid maps were calculated. The synthesized compounds were constructed as 3D structures, and their energies were minimized. The co-crystallized ligand was removed from the binding sites, allowing for the docking of the compounds discussed herein into those sites.

### 4.10. Pharmacokinetics and ADME analysis

ADME studies were performed utilizing the Swiss ADME platform, adhering to previously documented methodologies.^72^ These investigations were designed to evaluate physicochemical parameters^67^ to assess the drug-likeness and medicinal chemistry of the synthesized compounds, while also establishing correlations between biological activity and these physicochemical characteristics.

## 5. Statistics

GraphPad Prism 7 for Windows (Intuitive Software for Science, San Diego, CA, USA) was used to perform statistical analyses. Analysis of variance (ANOVA), a test for multiple comparisons, was employed to compare the experimental groups. A significant level of *P* < 0.05 was regarded as the cutoff point for identifying significant differences.

## 6. Conclusion

The findings from this study underscore the promising potential of novel camphor-derived heterocyclic compounds as effective anticancer agents, specifically targeting breast (MCF-7) and lung (A549) cancer cell lines. Notably, compound 20 emerged as a standout candidate, exhibiting exceptional inhibitory activity with an IC_50_ of 0.78 μM against MCF-7 cells and 1.69 μM against A549 cells. These values not only demonstrate its potency but also surpass the efficacy of established chemotherapeutics, including dasatinib (IC_50_ = 7.99 μM for MCF-7 and 11.8 μM for A549) and doxorubicin (IC_50_ = 3.10 μM for MCF-7 and 2.43 μM for A549).

Furthermore, the strong correlation between the high binding energy observed in molecular docking studies and the potent biological activity of compound 20 emphasizes the critical role of structural optimization in the design of new anticancer therapies. These results highlight the need for further exploration into the mechanisms of action and *in vivo* efficacy of compound 20, paving the way for its potential development as a therapeutic agent in cancer treatment. Continued research in this area could lead to effective new strategies for combating breast and lung cancers, ultimately improving patient outcomes.

## Ethical approval

This article does not include any studies that use human or animal tissues.

## Data availability

All data is available within the article.

## Author contribution

Z. H. and H. S., methodology, design experiments and writing draft manuscript. W. F. and W. H., supervision and editing manuscript. K. E., M. O. and G. H., molecular docking and biology.

## Conflicts of interest

The authors have no competing interests to declare that are relevant to the content of this article.
